# Relationship between retinal blood flow and cytokines in central retinal vein occlusion

**DOI:** 10.1186/s12886-020-01486-x

**Published:** 2020-06-05

**Authors:** Hidetaka Noma, Kanako Yasuda, Tatsuya Mimura, Akemi Ofusa, Masahiko Shimura

**Affiliations:** 1 Department of Ophthalmology, Hachioji Medical Center, Tokyo Medical University, 1163, Tatemachi, Hachioji, Tokyo, 193-0998 Japan; 2 Department of Ophthalmology, Teikyo University School of Medicine, Tokyo, Japan

**Keywords:** Central retinal vein occlusion, Laser speckle flowgraphy, Retinal blood flow, Placental growth factor, Soluble intercellular adhesion molecule-1, Interleukin-8

## Abstract

**Background:**

We evaluated the relationship between retinal blood flow and aqueous humor levels of cytokines/growth factors in patients with central retinal vein occlusion (CRVO).

**Methods:**

In an observational study, 64 eyes of 64 CRVO patients were examined before anti-vascular endothelial growth factor (VEGF) therapy. Blood flow was assessed in large vessels around and at the optic disk by determining the mean blur rate using laser speckle flowgraphy. Aqueous humor samples were obtained from the patients during anti-VEGF therapy and levels of the following molecules were measured by the suspension array method: soluble VEGF receptor (sVEGFR)-1, sVEGFR-2, VEGF, plancental growth factor (PlGF), platelet-derived growth factor (PDGF)-AA, soluble intercellular adhesion molecule (sICAM)-1, monocyte chemotactic protein (MCP)-1, interleukin (IL)-6, IL-8, IL-12(p70), and IL-13.

**Results:**

The mean blur rate of the affected eye was significantly lower than that of the unaffected eye. The mean blur rate showed a significant negative correlation with the log-transformed aqueous humor levels of PlGF, sICAM-1, and IL-8, but not VEGF.

**Conclusions:**

These findings suggest that retinal blood flow velocity might be more strongly correlated with inflammatory factors than VEGF in patients with nonischemic CRVO and macular edema.

## Background

Central retinal vein occlusion (CRVO) is a common condition that often causes macular edema, which is the most frequent reason for visual impairment in these patients. In CRVO, compression of the central retinal vein by an arteriosclerotic central retinal artery in the vicinity of the lamina cribrosa leads to venous thrombosis [1]. Thus, understanding the abnormalities of retinal hemodynamics underlying the pathogenesis of CRVO is critically important. Recently, it was reported that increased production of vascular endothelial growth factor (VEGF) is associated with macular edema in CRVO patients [2]. In addition, treatment with ranibizumab or aflibercept achieves significant improvement of visual acuity in CRVO patients with macular edema, supporting a role of VEGF in this condition [3, 4].

Measurement of ocular blood flow is commonly performed in various ophthalmic diseases, with both invasive and noninvasive methods being used to obtain blood flow data. We previously investigated the relationship between the blood flow velocity in the perifoveal capillaries and macular edema in patients with retinal vein occlusion by the tracing method using fluorescein angiography and a scanning laser ophthalmoscope [5, 6], but this method is invasive. On the other hand, several noninvasive techniques are available, including color Doppler imaging [7], laser Doppler velocimetry [8], and laser speckle flowgraphy (LSFG) [9–12]. Among these methods, LSFG is convenient for measuring blood flow in the clinical setting. Yamada et al. recently reported that the aqueous humor level of VEGF was correlated with the mean blur rate (MBR) in CRVO patients [13]. In addition to VEGF, we have found that intraocular levels of several inflammatory factors are significantly correlated with the severity of macular edema in CRVO patients [14]. However, the relation between intraocular levels of various factors/cytokines and MBR has not been clarified. Accordingly, we investigated the relation of MBR to aqueous humor levels of 11 cytokines or growth factors in patients with nonischemic CRVO receiving anti-VEGF therapy for macular edema.

## Methods

### Subjects

Our current research followed the tenets of the Declaration of Helsinki, with approval for this study obtained from the Ethics Committee of Tokyo Medical University Hachioji Medical Center. This manuscript does not include any individual participant details. This prospective observational study was conducted on 64 eyes of 64 patients with CRVO (30 females and 34 males) with no history of treatment. Each patient received intravitreal ranibizumab injection (IRI) at a dose of 0.5 mg in 0.05 ml (Lucentis; Genentech, Inc., South San Francisco, CA) or intravitreal aflibercept injection (IAI) at a dose of 2 mg in 0.05 ml (Eylea; Regeneron Pharmaceuticals, Inc., Tarrytown, NY, and Bayer HealthCare Pharmaceuticals, Berlin, Germany). The criteria for *IRI or IAI therapy* were a central macular thickness (CMT) > 300 μm and best-corrected visual acuity (BCVA) < 25/30. None of the patients had received treatment for macular edema before this study. The systolic blood pressure (SBP) and diastolic blood pressure (DBP) were measured with a mercury sphygmomanometer. Hypertension was diagnosed if patients were receiving antihypertensive drugs or had a blood pressure ≥ 140/90 mmHg.

After each patient gave informed consent, blood flow was measured in the large vessels at the optic disk and aqueous levels of cytokines were determined. LSFG was used to evaluate blood flow as the MBR [11, 13], while a mean volume of 0.1 mL of aqueous humor was collected by anterior chamber limbal paracentesis with a 30-gauge needle attached to an insulin syringe. *IRI or IAI therapy* was then administered through the pars plana at 3.5 mm from the limbus. The aqueous humor levels of cytokines were measured with enzyme-linked immunosorbent assays (Cat. No. HSCRMAG-32 K-02: sVEGFR1 and sVEGFR2; Cat. No. HCYTOMAG-60 K-08: VEGF, PDGF-AA, MCP-1, IL-6, IL-8, IL-12(p70) and IL-13; Cat. No. HCVD1MAG-67 K-01: PlGF; Cat. No. HCVD2MAG-67 K-01: sICAM-1) according to the manufacturer’s instructions (Funakoshi Corporation Ltd., Tokyo, Japan) [14]. We obtained informed consent for aqueous humor puncture from patients, as well as IRB approval. BCVA was determined as the logarithm of the minimum angle of resolution (LogMAR). Exclusion criteria were a history of glaucoma, uveitis, retinal diseases other than CRVO, diabetes mellitus, rubeosis iridis, ocular infection, laser photocoagulation, intraocular surgery (including cataract surgery), and conditions causing difficulty with measurement of MBR (cataract with severe opacity, vitreous hemorrhage, inadequate mydriasis, or corneal opacity). Ischemic CRVO was defined as more than 10 disc areas of nonperfusion on fluorescein angiography according to the Central Retinal Vein Occlusion Study Group [15], and patients with ischemic CRVO were also excluded.

### Laser speckle flowgraphy

The LSFG system used in this study (LSFG-NAVI; Softcare Co, Ltd., Fukuoka, Japan) has been described previously [11, 13]. In brief, light reflected from the fundus produces a speckled pattern in the plane where the area sensor is focused, while light reflected by moving erythrocytes causes blurring of the speckle pattern [16]. A fundus camera equipped with an 830 nm diode laser and a charge-coupled device sensor (750 × 360 pixels) were used to obtain images of the speckle contrast pattern produced by interference as laser light was scattered by red blood cells moving through vessels in the ocular fundus. Images were acquired continuously at 30 frames/sec over a 4-s period and then averaged to produce a composite map of ocular blood flow.

**Fig. 1:**
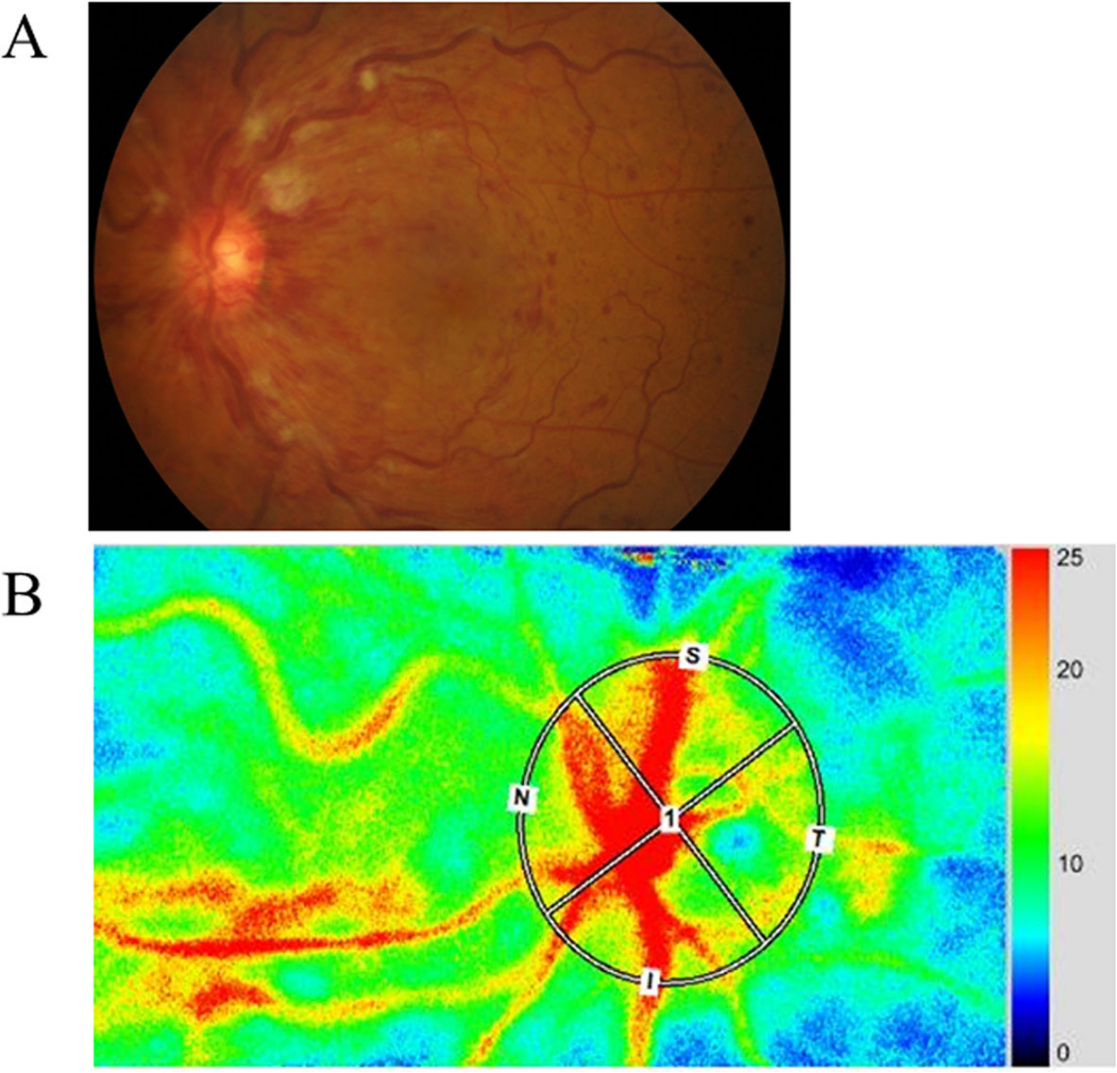
Representative fundus color photograph and representative MBR data obtained with LSFG. **a** Fundus color photograph shows central retinal vein occlusion. **b** A circle was manually using an elliptical rubber band to delineate the disc rim. Red and blue indicate faster and slower blood flow, respectively

The MBR thus determined was expressed in arbitrary units (AU) and displayed as a 2-dimensional color-coded map of blood flow velocity. After manually setting a circle around the optic disc by using a rubber band to delineate the disc rim, we investigated the MBR of the major vessels (arteries and veins) within this region (Fig. 1). We evaluated the microcirculation in the head of the optic nerve by measuring the MBR of the optic disc, as reported previously [17]. We fitted an elliptical band around the optic disc (Fig. 1), so measurements of MBR within the region were not affected by the vessel tortuousness. Therefore, we were able to measure the MBR in all cases. Since the MBR in the vascular area includes choroidal blood flow, we subtracted the mean MBR of the tissue area from the mean MBR of the vascular area [18] to evaluate blood flow in the retinal vessels without the influence of choroidal flow. All measurements were performed in triplicate and the mean MBR value was calculated. Eye positions were recorded by performing LSFG with an auto tracking function, making it possible to capture the same area again during subsequent examinations with high reproducibility. There were no comorbidities in the unaffected eyes.

### Statistical analysis

All results are expressed as the mean ± standard deviation, as the median with interquartile range, or as frequencies. Paired *t*-test was employed to compare normally distributed continuous variables. To investigate the relations among cytokines, inflammatory factors, SBP, DBP, and MBR, Spearman’s rank-order correlation coefficients or Pearson’s correlation coefficients were calculated. Normality of data was assessed using the Shapiro–Wilk test. Statistical significance was considered at *P* < 0.05.

## Results

**Fig. 2:**
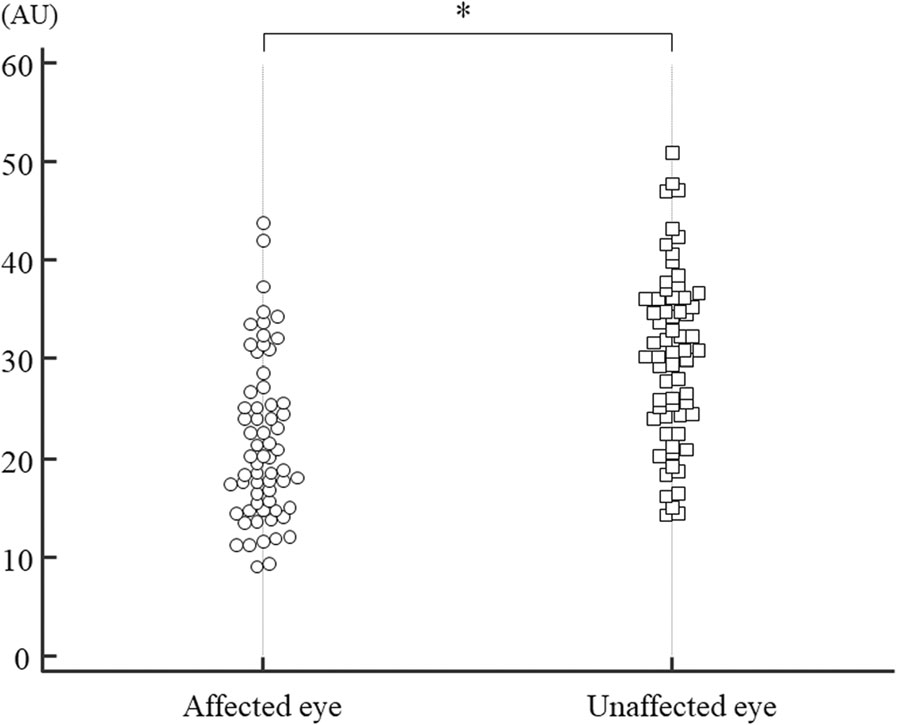
Mean blur rate of the affected and unaffected eyes. The mean blur rate was significantly lower in the affected eye than the unaffected eye (21.7 ± 8.1 AU vs. 30.4 ± 8.7 AU, *P* < 0.001)

The clinical characteristics of the 64 CRVO patients with macular edema are summarized in Table 1. They included 30 women and 34 men aged 69.2 ± 10.6 years (mean ± SD). The mean duration of macular edema was 35.6 ± 27.3 days (range: 10–120 days). The duration of macular edema was defined as the estimated loss of visual acuity due to intravitreal treatment. Fifty patients (78.1%) had hypertension and 27 patients (42.2%) had hyperlipidemia. At the initial examination, mean BCVA was logMAR 0.57 ± 0.37 and mean CMT was 716 ± 192 μm. MBR was significantly lower in the affected eye than in the unaffected eye (21.7 ± 8.1 AU vs. 30.4 ± 8.7 AU, *P* < 0.001) (Fig. 2) (paired *t*-test). There was no significant difference between MBR at baseline (21.7 ± 8.1 AU) and 1 month after intravitreal treatment (22.0 ± 9.2 AU) (*P* = 0.775) (paired *t*-test). There was no correlation between MBR and SBP or DBP (r = 0.17, *P* = 0.186 and r = − 0.01, *P* = 0.991, respectively) (Pearson’s correlation coefficient). However, significant correlations were noted between MBR and BCVA, and between MBR and CMT (r = − 0.37, *P* = 0.003 and r = − 0.27, *P* = 0.030, respectively) (Pearson’s correlation coefficient).There were also no correlations between the mean duration of macular edema and the aqueous humor levels of sVEGFR-1, sVEGFR-2, VEGF, PlGF, PDGF-AA, sICAM-1, MCP-1, IL-6, IL-8, IL-12 (p70), or IL-13 (r = 0.01, *P* = 0.919, r = − 0.09, *P* = 0.456, r = − 0.03, *P* = 0.821, r = − 0.24, *P* = 0.054, r = − 0.10, *P* = 0.433, r = − 0.14, *P* = 0.243, r = − 0.18, *P* = 0.141, r = 0.06, *P* = 0.593, r = − 0.11, *P* = 0.351, r = − 0.08, *P* = 0.508, and r = 0.01, *P* = 0.984, respectively) (Spearman’s rank-order correlation coefficient or Pearson’s correlation coefficient).

**Table 1:** Clinical and demographic data of the CRVO patients

	CRVO
No. (Female/Male)	64 (30/34)
Age, years (mean ± SD)	69.2 ± 10.6
Hypertension (*n*)	50 (78.1%)
Systolic blood pressure (mmHg)	144 ± 14
Diastolic blood pressure (mmHg)	70 ± 12
Hyperlipidemia (*n*)	27 (42.2%)
Duration of CRVO, days (mean ± SD)	35.6 ± 27.3
BCVA, logMAR (mean ± SD)	0.57 ± 0.37
Foveal thickness, μm (mean ± SD)	716 ± 192
Vessels for MBR measurement (number/eye)	5.0 ± 1.4

*CRVO* Central retinal vein occlusion, *SD* Standard deviation, *No.* Number of eyes, *BCVA* Best-corrected visual acuity, *logMAR* Logarithm of minimal angle of resolution; MBR, mean blur rate

**Fig. 3:**
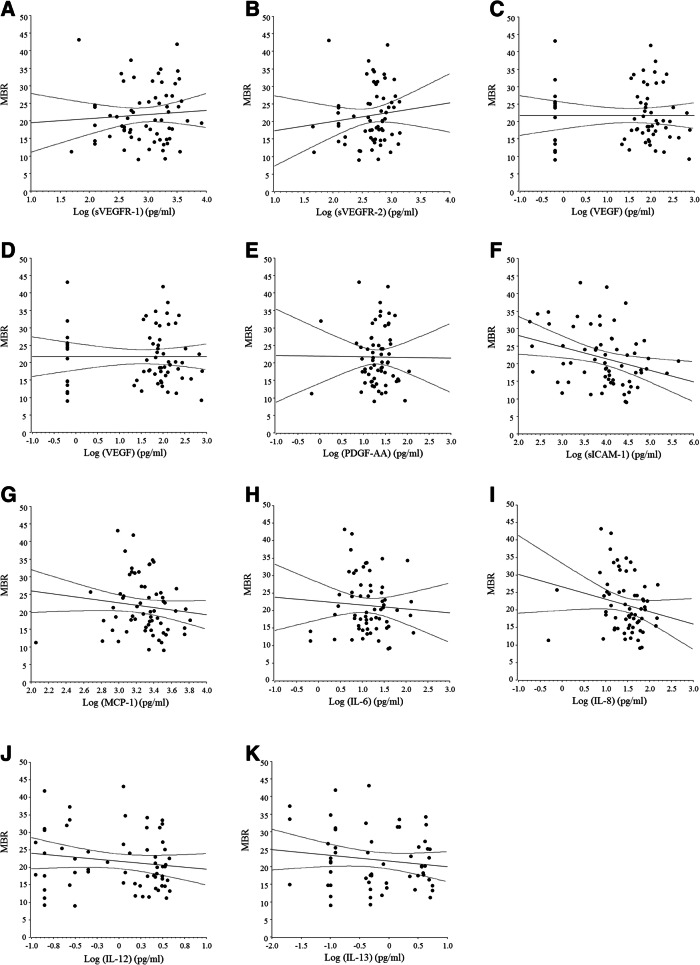
Correlations between the mean blur rate and the log-transformed aqueous humor levels of cytokines, growth factors, and receptors. **a** MBR was not significantly correlated with the log-transformed aqueous level of sVEGFR-1 (r = 0.07, *P* = 0.577). **b** MBR was not significantly correlated with the log-transformed aqueous level of sVEGFR-2 (r = 0.11, *P* = 0.375). **c** MBR was not significantly correlated with the log-transformed aqueous level of VEGF (r = − 0.01, *P* = 0.996). **d** MBR showed a significant negative correlation with the log-transformed aqueous level of PlGF (r = − 0.27, *P* = 0.031). **e** MBR was not significantly correlated with the log-transformed aqueous level of PDGF-AA (r = − 0.01, *P* = 0.952). **f** MBR showed a significant negative correlation with the log-transformed aqueous level of sICAM-1 (r = − 0.31, *P* = 0.019). **g** MBR was not significantly correlated with the log-transformed aqueous level of MCP-1 (r = − 0.18, *P* = 0.152). **h** MBR was not significantly correlated with the log-transformed aqueous level of IL-6 (r = − 0.06, *P* = 0.615). **i** MBR showed a significant negative correlation with the log-transformed aqueous level of IL-8 (r = − 0.27, *P* = 0.033). **j** MBR was not significantly correlated with the log-transformed aqueous level of IL-12 (p70) (r = − 0.15, *P* = 0.252). **k** MBR was not significantly correlated with the log-transformed aqueous level of IL-13 (r = − 0.14, *P* = 0.285)

Baseline aqueous humor levels of the 11 molecules tested (growth factors, cytokines, and receptors), including sVEGFR-1, sVEGFR-2, VEGF, PlGF, PDGF-AA, sICAM-1, MCP-1, IL-6, IL-8, IL-12 (p70), and IL-13, are listed in Table 2. MBR showed a significant negative correlation with the log-transformed levels of PlGF, sICAM-1, and IL-8 (r = − 0.27, *P* = 0.031, r = − 0.31, *P* = 0.019, and r = − 0.27, *P* = 0.033, respectively) (Fig. 3) (Pearson’s correlation coefficient). On the other hand, MBR was not significantly correlated with the log-transformed levels of sVEGFR-1, sVEGFR-2, VEGF, PDGF-AA, MCP-1, IL-6, IL-12 (p70), or IL-13 (Fig. 3) (Pearson’s correlation coefficient).

**Table 2:** Aqueous Humor Factors/Cytokines at Baseline

Factors/Cytokines	Baseline
sVEGFR-1 (pg/ml)	1129 [391–2082]
sVEGFR-2 (pg/ml)	520 [360–728]
VEGF (pg/ml)	77.0 [28.7–129]
PlGF (pg/ml)	6.82 [2.66–13.4]
MCP-1 (pg/ml)	2169 [1335–2900]
sICAM-1 (pg/ml)	10,100 [3180–28,100]
PDGF-AA (pg/ml)	21.8 [14.9–33.3]
IL-6 (pg/ml)	12.3 [6.39–27.6]
IL-8 (pg/ml)	32.8 [15.1–54.5]
IL-12 (pg/ml)	1.62 [0.27–2.90]
IL-13 (pg/ml)	0.50 [0.12–3.60]

*sVEGFR* Soluble vascular endothelial growth factor receptor, *VEGF* Vascular endothelial growth factor, *PlGF* Placental growth factor, *MCP* Monocyte chemotactic protein, *sICAM* Soluble intercellular adhesion molecule, *PDGF* Platelet-derived growth factor, *IL* Interleukin

## Discussion

There were two main findings of this study performed in patients with nonischemic CRVO and macular edema. (1) The MBR of the affected eye was significantly lower than the MBR of the unaffected eye. (2) MBR showed a significant negative correlation with the log-transformed aqueous humor levels of PlGF, sICAM-1, and IL-8. These findings suggest that retinal blood flow velocity might be more strongly correlated with levels of inflammatory factors than VEGF in patients with nonischemic CRVO and macular edema.

This study demonstrated that the MBR of the affected eye was significantly lower than that of the unaffected eye, although we excluded patients with ischemic CRVO because they can potentially develop neovascular glaucoma and require pan-retinal photocoagulation. A number of previous studies have also demonstrated that blood flow velocity is lower in eyes with CRVO than in normal eyes [13, 19–21], corresponding to our finding.

In addition, we identified a significant correlation between the decrease of MBR and a higher aqueous humor level of sICAM-1, suggesting that sICAM-1 influences the blood flow velocity in patients with nonischemic CRVO and macular edema. Expression of ICAM-1 has been demonstrated in the retinal vascular endothelium, retinal pigment epithelium, and choroid by in vivo and in vitro studies, and ICAM-1 is also expressed by leukocytes migrating into the retina [22]. It was reported that upregulation of ICAM-1 expression induces retinal leukostasis, with increased rolling and adhesion of leukocytes to vessel walls that leads to stagnation [23]. It was also reported that retinal vein occlusion in vivo increases leukocyte rolling and adhesion to the vein walls, resulting in stagnant blood flow [24]. Thus, entrapment of leukocytes associated with increased rolling and adhesion of these cells may reduce the MBR in patients with nonischemic CRVO and macular edema.

This study also demonstrated a significant negative correlation between MBR and the aqueous humor levels of PlGF and IL-8. PlGF is a member of the VEGF family [25, 26], and it promotes the proliferation of endothelial cells [27] and enhances leukocyte chemotaxis [27, 28]. PlGF binds exclusively to VEGFR-1 [29] and shows higher affinity for this receptor than VEGF [30]. On the other hand, IL-8 is a potent chemoattractant that activates neutrophils and T cells. Production of IL-8 is induced by exposure of vascular endothelial cells to hypoxia and oxidative stress [31–33], and it was reported that IL-8 promotes the adhesion of leukocytes to the vascular endothelium [34, 35]. Thus, PIGF and IL-8 could promote chemotaxis and adhesion of leukocytes that reduces the MBR in CRVO patients with macular edema. We previously reported that the aqueous humor levels of PlGF, sICAM-1, and IL-8 were significantly correlated with each other in CRVO patients [14]. In addition, the mean duration of macular edema was unrelated to the levels of 3 cytokines negatively correlated with the MBR. This suggests that the negative correlations of these cytokines were probably not just due to the duration of symptoms, making it possible that the three factors act together to reduce MBR in patients with nonischemic CRVO and macular edema.

Unexpectedly, we could not identify a significant correlation between MBR and the log-transformed aqueous humor level of VEGF, although Yamada et al. [13] previously reported such a correlation. These differing results were probably obtained because we excluded patients with ischemic CRVO. Since VEGF levels are very high in ischemic CRVO, it may be easier to demonstrate a relation with MBR. Williamson and Baxter [36] reported that a minimum blood velocity < 3.0 cm/sec was highly predictive of the development of iris neovascularization in patients with CRVO. Taken together with these reports, our findings suggest that MBR might be more strongly influenced by inflammatory factors than VEGF in patients with nonischemic CRVO and macular edema. However, Yamada and colleagues reported a strong correlation of flow parameters (MBR and arteriovenous passage time) with VEGF levels and a lower affected/healthy eye MBR ratio in ischemic CRVO [13]. Thus, MBR and various cytokines may be correlated along with higher VEGF levels in ischemic patients. Based on the present findings, it is possible that elevation of the intravascular pressure due to malperfusion drives cystoid macular edema, while consequent leakage of blood compounds into the ocular tissues induces an inflammatory response with elevation of various cytokines that exacerbates this condition. Therefore, further data are required to more fully reveal the relationship between MBR and cytokines.

We previously reported that there are significant differences in the vitreous fluid levels of VEGF, sICAM-1, and IL-6 between nonischemic and ischemic cases of CRVO, as well as a significant correlation between the flare value (as an index of inflammation) and vitreous levels of VEGF, sICAM-1, and IL-6 in patients with CRVO [37]. Those previous findings suggested that ischemia and inflammation are both related to the pathogenesis of ischemic CRVO, while inflammation is related more to the pathogenesis of nonischemic CRVO. Furthermore, the flare value correlated more closely with the vitreous level of VEGF than with inflammatory factors in patients with CRVO [37], so anti-VEGF therapy seems to be more effective than anti-inflammatory agents not only in ischemic CRVO but also in nonischemic CRVO. However, inflammation is more closely related to the pathogenesis of nonischemic CRVO, so anti-inflammatory agents are also considered effective in nonischemic CRVO. The efficacy of anti-inflammatory agents in nonischemic CRVO also warrants clinical investigation.

The present study had the following limitations. While we found no significant correlation between MBR and VEGF, this might have been due to marked variation of VEGF values among patients or unknown confounders. Unfortunately, it is difficult to obtain data on aqueous humor cytokine levels at multiple time points because sampling cannot be done ethically in patients without recurrence of macular edema. Further attempts will be required to measure aqueous humor cytokine levels over time in future studies. Furthermore, this study was a cross-sectional study with a focus on the relationship between retinal blood flow and cytokine levels. Therefore, we did not investigate the relationship between MBR and reaction to anti-VEGF therapy in this study; this relationship will need to be confirmed in future studies. In addition, the clinical pictures of nonischemic and ischemic CRVO are generally quite different [38], which is why we excluded patients with ischemic CRVO from this study. As a result, patients with neovascular glaucoma, panretinal photocoagulation, and severe loss of visual acuity were also excluded. Further studies are be required to compare the retinal blood flow and levels of cytokines between nonischemic and ischemic CRVO.

## Conclusions

MBR was significantly lower in the affected eye than the unaffected eye in patients who had nonischemic CRVO and macular edema. MBR showed a significant negative correlation with the log-transformed aqueous humor levels of PlGF, sICAM-1, and IL-8, but not VEGF. Our findings suggest that MBR might be better correlated with inflammatory factors such as sICAM-1 than VEGF in patients who have nonischemic CRVO and macular edema.

## Data Availability

Data will not be shared because the authors are performing other analyses that have not yet been published.

## Abbreviations

AU: Arbitrary units
BCVA: Best-corrected visual acuity
CMT: Central macular thickness
CRVO: Central retinal vein occlusion
DBP: Diastolic blood pressure
IAI: Intravitreal aflibercept injection
IL: Interleukin
IRI: Intravitreal ranibizumab injection
LSFG: Laser speckle flowgraphy
MBR: Mean blur rate
MCP: Monocyte chemotactic protein
PDGF: Platelet-derived growth factor
PlGF: Plancental growth factor
sICAM: Soluble intercellular adhesion molecule
SBP: Systolic blood pressure
sVEGFR: Soluble vascular endothelial growth factor receptor
VEGF: Vascular endothelial growth factor
